# Immunophenotypic characterization and clinical outcome in cats with lymphocytosis

**DOI:** 10.1111/jvim.15650

**Published:** 2019-11-06

**Authors:** Emily D. Rout, Julia D. Labadie, Kaitlin M. Curran, Janna A. Yoshimoto, Anne C. Avery, Paul R. Avery

**Affiliations:** ^1^ Department of Microbiology, Immunology and Pathology, College of Veterinary Medicine and Biomedical Sciences Colorado State University Fort Collins Colorado; ^2^ Department of Clinical Sciences, Carlson College of Veterinary Medicine Oregon State University Corvallis Oregon

**Keywords:** clonality, feline, flow cytometry, leukemia, polymerase chain reaction for antigen receptor rearrangements

## Abstract

**Background:**

Lymphocytosis is relatively common in cats, but few studies describe lymphocyte populations or the clinical course associated with different immunophenotypic expansions.

**Hypothesis/Objectives:**

We hypothesized that cats frequently develop non‐neoplastic lymphocytosis and that different neoplastic immunophenotypes have variable prognoses. We aimed to characterize the lymphocyte expansions in a large population of cats with lymphocytosis and to assess clinical presentation and outcome in a subset.

**Animals:**

Three cohorts of cats older than 1 year with lymphocytosis (>6000/μL) were examined to define immunophenotypic categories (n = 146), evaluate outcome (n = 94), and determine prevalence of immunophenotypes (n = 350).

**Methods:**

Retrospective study of cats with blood submitted for flow cytometry. Medical records (n = 94) were reviewed for clinical data, treatment, and survival information.

**Results:**

Five major immunophenotypic categories were identified: B cell, heterogeneous (≥2 lineages expanded), CD4+ T cell, CD4−CD8− (double negative [DN]) T cell, and CD5‐low‐expressing T cell. B‐cell and heterogeneous phenotypes were more consistent with a non‐neoplastic process, having polyclonal antigen receptor gene rearrangements, younger age at presentation, lower lymphocyte counts, and prolonged survival. The neoplastic phenotypes, CD4+ T cell, DN T cell, and CD5 low T cell, had different median survival times (752 days [n = 37], 271 days [n = 7], 27.5 days [n = 12], respectively). Among CD4+ T‐cell cases, cats with abdominal lymphadenopathy, intestinal involvement, or both and females had shorter survival. Among 350 cats with lymphocytosis, CD4+ T‐cell lymphocytosis was most common, followed by heterogeneous and B‐cell phenotypes.

**Conclusions and Clinical Importance:**

Neoplastic CD4+ T‐cell lymphocytosis is common in cats and has a prolonged clinical course compared to aberrant T‐cell phenotypes. Cats with heterogeneous and B‐cell lymphocyte expansions commonly have non‐neoplastic disease.

**Abbreviations**
CLL: chronic lymphocytic leukemia
CSU‐CI: Colorado State University‐Clinical Immunology
DN: double negative
FeLV: feline leukemia virus
IG: immunoglobulin
IGH‐VDJ: immunoglobulin heavy chain variable (V)‐diversity (D)‐joining (J) gene rearrangement
IMHA: immune‐mediated hemolytic anemia
IQR: interquartile range
LGL: large granular lymphocytic
MST: median survival time
PARR: PCR for antigen receptor rearrangements
PBMC: peripheral blood mononuclear cells
PE: physical examination
TRG: T‐cell receptor gamma

## 1 INTRODUCTION

Lymphocytosis is relatively common in cats, but few studies have examined its flow cytometric features. Lymphocytosis may be attributed to non‐neoplastic or neoplastic processes. Our goals were to determine how frequently lymphocytosis in cats is neoplastic, and among cases of neoplastic lymphocytosis, the utility of flow cytometry to characterize clinically relevant phenotypes.

Few studies have described non‐neoplastic expansions of peripheral lymphocytes in cats, which may be attributed to physiologic or inflammatory processes. Absolute lymphocytosis has been documented with a number of infections, including *Cytauxzoon felis, Toxoplasma gondii, Aelurostrongylus abstrusus, Leishmania infantum, Mycoplasma haemofelis, Bartonella henselae*, feline leukemia virus (FeLV), and feline immunodeficiency virus.^1^, ^2^, ^3^, ^4^, ^5^, ^6^, ^7^, ^8^, ^9^ Peripheral lymphocytosis was reported in several studies of primary immune‐mediated hemolytic anemia (IMHA), and bone marrow lymphoid hyperplasia has been described in IMHA and pure red cell aplasia cases.^10^, ^11^, ^12^, ^13^, ^14^ Hyperthyroidism and treatment with carbimazole or methimazole were reported to result in lymphocytosis in a small subset of cats.^15^, ^16^, ^17^ Lymphocytosis also was present in a small number of cats with hypoadrenocorticism.^18^, ^19^

Lymphocytosis attributed to a neoplastic process may be seen in cases of leukemia or cases of lymphoma with circulating neoplastic lymphocytes. Clinical findings and outcome have been reported for 18 cats with clinically defined chronic lymphocytic leukemia (CLL).^20^ Chronic lymphocytic leukemia was defined as >9000 small, mature lymphocytes/μL with flow cytometry or polymerase chain reaction (PCR) for antigen receptor rearrangements (PARR) confirmation or both and evidence of a peripheral cytopenia or >15% lymphocytes in the bone marrow or both. Almost all these cats had an expanded T‐cell population (17/18), and most (16/17) were of CD4+ T‐cell origin.^20^ The clinical course appeared relatively indolent with most cats treated with prednisone and chlorambucil. Six cases of acute lymphoblastic leukemia were described in cats, diagnosed based on the percentage of blasts and concurrent cytopenias, and found a short median survival (55 days).^21^ Immunophenotyping by PARR, flow cytometry, immunohistochemistry, or some combination of these identified 4 cases as B‐cell lineage and 1 case as T‐cell lineage, and 1 case expressed B‐cell and T‐cell antigens. The frequency with which cats develop peripheral lymphocytosis secondary to lymphoma is unclear, because many case series of lymphoma in cats do not specifically discuss peripheral lymphocyte counts. In 3 studies representing several forms of lymphoma, small numbers of cases had high peripheral lymphocyte counts, reaching 78 600 cells/μL.^22^, ^23^, ^24^ Several studies have shown that large granular lymphocytic (LGL) lymphoma can have circulating neoplastic lymphocytes, but a recent large study suggests absolute lymphocytosis is not common with this subtype.^25^, ^26^, ^27^, ^28^

Our experience is that cats present more commonly with ≥2 expanded subsets of peripheral lymphocytes than do dogs. How often these cases are non‐neoplastic in nature has not been determined, and in cases where the cells are neoplastic, the prognostic value of immunophenotyping is unclear. We performed a retrospective study to examine the range of phenotypes in cats presenting with lymphocytosis without any a priori definitions of disease. We examined clonality, clinical presentation, and outcome on a subset of cases.

## 2 MATERIALS AND METHODS

### 2.1 Study population

A retrospective study was performed using feline blood samples submitted to the Colorado State University‐Clinical Immunology (CSU‐CI) laboratory for immunophenotyping by flow cytometry. All cats included in the study were older than 1 year of age and had >6000 lymphocytes/μL (the upper limit of the lymphocyte reference interval for cats at our institution). Three cohorts of patients were examined for different components of this study: (i) definition cohort; (ii) outcome cohort; and (iii) concordance cohort.

#### 2.1.1 Definition cohort

An initial study population of 146 cats was examined to define immunophenotypic categories. This population included all cats that had both flow cytometry and PARR performed on blood samples submitted between October 24, 2014, and November 16, 2017. In some cases (25%), concurrent flow cytometry and PARR were requested by the veterinarian at the time of submission, but in many cases, PARR (75%) was performed after an equivocal flow cytometry result.

#### 2.1.2 Outcome cohort

A separate study population of 94 cats with outcome data was examined, which included cats that had immunophenotyping on blood performed between July 12, 2006, and April 20, 2010. Veterinary clinics were contacted for medical records and clinical data were collected by reviewing records. All cats included in this cohort had persistent or recurrent lymphocytosis as described above identified on ≥2 separate CBCs.

#### 2.1.3 Concordance cohort

A third study population of 350 cats was examined to determine the prevalence of immunophenotypes in a larger cohort of animals and to determine whether the outcome cohort was representative of a larger population. This population included all cats that had blood samples submitted for flow cytometry between January 1, 2017, and December 1, 2017.

### 2.2 Clinical variables

For all samples, signalment, physical examination (PE) findings, and laboratory findings were provided by the submitting veterinarian on the standard CSU‐CI laboratory submission form. Complete blood count data from the time of submission were used to identify hematologic abnormalities. Anemia was defined as a hematocrit <28%, thrombocytopenia by a platelet total <200 000/μL with no clumps noted, and neutropenia as <2000 neutrophils/μL.

Additional information was gathered for the 94 cats in the outcome cohort. Medical records were provided by the submitting veterinarians, which represented non‐referral and referral clinics distributed across 24 states. Clinical signs and concurrent diseases were abstracted from the medical history. Lymph nodes, spleen, liver, and intestine were considered abnormal if enlargement, masses, or thickening were identified by palpation, ultrasound examination, or radiology. Overall survival was calculated from the time of flow cytometry submission, because this date was available for all cases, to the time of death due to any cause. In many cases, the exact cause of death was not known. Overall survival additionally was calculated from the date of onset of lymphocytosis, but this information was not available for all cases. Cats alive at the time of data collection or lost to follow‐up were censored at the last date of contact.

### 2.3 Flow cytometry

Flow cytometry was performed on blood samples as detailed in the Supporting Information, using a panel consisting of antibodies recognizing canine CD21, canine CD18, and feline CD5, CD4, and CD8. Total cell counts were determined for CD4+ T cells, CD8+ T cells, total CD5+ T cells, and CD21+ B cells by multiplying the percentage of each subset within the gated lymphocyte population by the total lymphocyte count on the CBC. Subset cell counts for each case were compared to internally generated reference intervals to identify subset expansions. Our laboratory reference interval upper limits for feline blood are 3300 CD4+ T cells/μL, 1800 CD8+ T cells/μL, 5400 CD5+ T cells/μL, and 1700 CD21+ B cells/μL. The percentage of CD4−CD8− T cells was calculated by determining the percentage of CD5+ T cells that did not express CD4 or CD8 antigens. Among 10 control cats with normal peripheral lymphocyte counts and no histologic or flow cytometric evidence of lymphoproliferative disease, the median percentage of CD4−CD8− T cells was 22.6% (median total count, 269 cells/μL), suggesting minor populations of CD4−CD8− T cells are not uncommon in healthy cats.

### 2.4 PCR for antigen receptor rearrangements

Polymerase chain reaction for antigen receptor rearrangements was performed on DNA extracted from blood samples, as previously described,^29^ to assess clonality of immunoglobulin (IG) and T‐cell receptor gamma (TRG) gene rearrangements. All 146 cases in the definition cohort and a subset of cases in the outcome cohort had PARR with primers targeting complete IG heavy chain V‐D‐J (IGH‐VDJ) and TRG gene rearrangements. A subset of cases in the definition cohort with B‐cell expansions had expanded PARR targeting additional IG gene rearrangements: incomplete immunoglobulin heavy chain diversity (D)‐joining (J) gene rearrangements, kappa deleting element rearrangements, and IG lambda light chain rearrangements.

We previously determined that our IGH‐VDJ PARR reaction only detected 50% of feline B‐cell neoplasms, but targeting additional IG gene rearrangements increased B‐cell PARR sensitivity from 50% to 87%.^29^ The sensitivity of our TRG PARR primers to detect clonality in T‐cell neoplasms was 97%.

### 2.5 Statistical analysis

For all cases in the outcome cohort, continuous (age, cell counts) and categorical variables were summarized and descriptive statistics calculated. Cats in each immunophenotypic category were compared. For continuous variables, an omnibus Kruskal‐Wallis test was calculated; if significant, Dunn's test subsequently was calculated. For categorical variables, Fisher's exact test was used with subsequent pairwise tests if significant. Individuals with missing or unknown data were excluded from analysis. Log‐rank tests were used to compare survival of the immunophenotypic categories from the time of flow cytometry, adjusting for multiple comparisons. Survival also was calculated using the date of lymphocytosis onset as a sensitivity analysis. Among CD4+ T‐cell cases, a log‐rank test was used to determine whether age, sex, hematologic variables, PE findings, and treatment affected survival time. Multivariable survival analysis was not performed because of inadequate sample size. Continuous risk factors were categorized into groups above and below the median for survival analysis. Treatment protocols were variable across cases, and thus treatment was categorized as (i) none; (ii) PO: corticosteroid, chlorambucil, or both; (iii) injectable/multi‐agent chemotherapy: single‐agent injectable chemotherapy, or multi‐agent chemotherapy. Corticosteroid treatment included prednisone or prednisolone, and in many cases, it was not clear which of these 2 drugs was used. Corticosteroid only and corticosteroid/chlorambucil combination treatment were combined for analysis because there was no difference in outcome between these groups. Phenotype distribution, age, sex, hematologic variables, and PE findings were compared between the outcome and concordance cohorts using Fisher's exact and Kruskal‐Wallis tests. All statistical analysis was performed in R version 3.3.2, and a *P*‐value <.05 was considered significant.

## 3 RESULTS

### 3.1 Flow cytometric characterization

**Figure 1:**
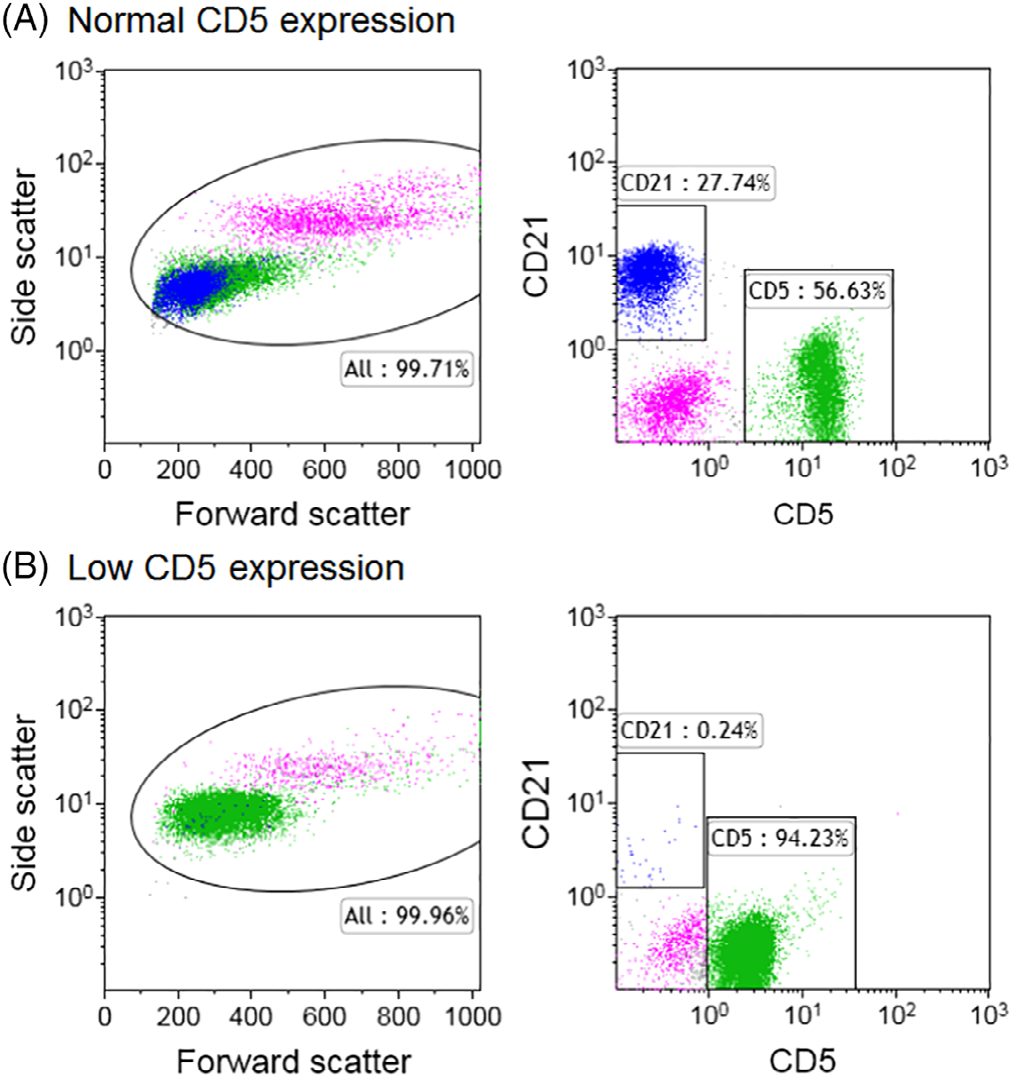
Representative flow cytometry plots from blood comparing normal CD5 expression (A) to low CD5 expression (B). Flow cytometry plots from a heterogeneous case with normal CD5 expression (A) and a CD5 low T‐cell case (B) are presented. Size plots (left) with forward scatter on the horizontal axis and side scatter on the vertical axis show CD18^high^ cells (neutrophils, monocytes) in pink, CD5+ T‐cells in green, and CD21+ B‐cells in blue. Fluorescence dot plots (right) demonstrate the level of CD5 expression on the horizontal axis. Cases were classified as CD5 low T cell when the entire neoplastic T‐cell population was situated in the second decade (10^0^‐10^1^) (B, right). Nonviable cells and platelets are excluded from analysis by propidium iodide staining and CD61 expression, respectively (not shown)

One hundred forty‐six cases (definition cohort) with flow cytometry and PARR results were reviewed to define phenotypic categories. Cases with sole subset expansions of CD21+ lymphocytes were classified as B cell, of CD8+ T cells as CD8+ T cell, and of CD4+ T cells as CD4+ T cell. When ≥2 of the subsets were expanded, the case was classified as heterogeneous. Cases in which CD4−CD8−CD5+ T cells made up ≥50% of all CD5+ T cells were classified as double negative (DN) T cell. Cases with low surface expression of the CD5 antigen by flow cytometry were classified as CD5 low T cell. Expression of CD5 was considered low if the fluorescence intensity of the entire T‐cell population was contained in the second decade on a log scale, rather than the third decade (Figure 1). As described below, low CD5 expression was associated with poor outcome, and any case with low CD5 expression was classified as CD5 low T cell, regardless of whether there was a concurrent CD21+ B‐cell expansion. Additionally, there was no difference in clinical presentation or overall survival based on CD4 or CD8 antigen expression within the CD5 low T‐cell group, and these cases were grouped together for the study.

### 3.2 Clonality

**Figure 2:**
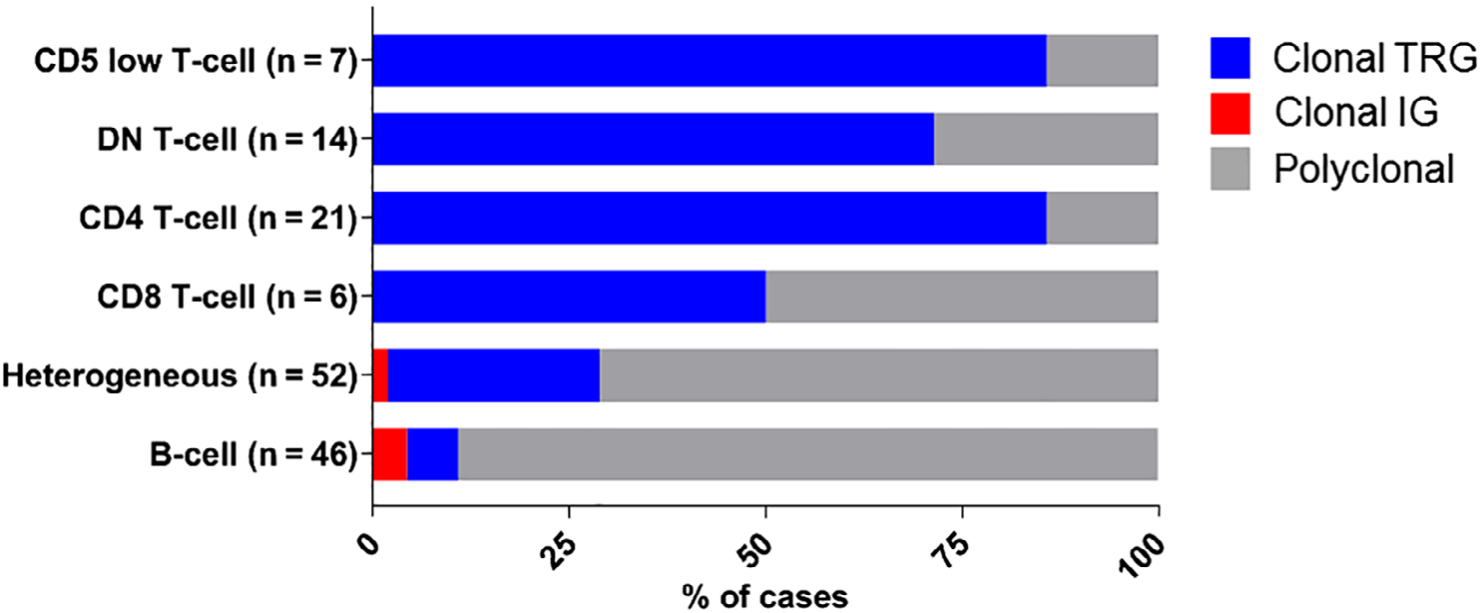
Polymerase chain reaction for antigen receptor rearrangements (PARR) results for 146 cats with different immunophenotypes. The percent of cases with a clonal T‐cell receptor gamma (TRG) (blue), clonal immunoglobulin (IG) (red), or polyclonal rearrangements (gray) within a phenotypic group are depicted. The total number of cases (n) in each phenotypic group is provided. Of note, 81% of cases in the combined CD5 low T‐cell, DN T‐cell, and CD4+ T‐cell groups had clonal TRG gene rearrangements; 50% of the CD8+ T‐cell cases were clonal; 71% of heterogeneous and 89% of B‐cell cases had polyclonal IG and TRG gene rearrangements

The PARR results were available for all 146 cats in the definition cohort. The majority of CD4+ T‐cell cases (86%), DN T‐cell cases (71%), and CD5 low T‐cell cases (86%) had clonal TRG rearrangements, indicating a neoplastic process (Figure 2). Half of the CD8+ T‐cell cases had a clonal TRG rearrangement and half were polyclonal, suggesting the CD8+ T‐cell phenotype may be seen with neoplastic and non‐neoplastic processes, although the overall frequency of this phenotype was quite low. The 3 clonal CD8+ T‐cell cases had higher proportions of CD8+ T cells in the lymphocyte population (>80%), compared to non‐clonal CD8+ T‐cell cases (44%‐63%), suggesting the proportion of CD8+ T cells may be useful in predicting clonality. The majority of B‐cell cases (94%) and heterogeneous cases (73%) had polyclonal TRG rearrangements.

All 146 cats in the definition cohort had IG PARR with IGH‐VDJ primers. All CD4+ T‐cell cases, DN T‐cell cases, and CD5 low T‐cell cases had polyclonal IG rearrangements. One B‐cell case and 1 heterogeneous case had clonal IG rearrangements with IGH‐VDJ primers. We performed expanded PARR using additional IG primer sets in 92 of 98 B‐cell and heterogeneous cases where DNA was still available. The 2 clonal IG cases originally detected remained clonal, and 1 new clonal B‐cell case was detected with kappa deleting element primers. Overall, only 2 of 46 B‐cell cases and 1 of 52 heterogeneous cases had a clonal IG rearrangement (Figure 2). One clonal B‐cell case had 30 535 CD21+ cells/μL and histologic confirmation of lymphoma in the spleen, kidney, intestine, and liver. The second B‐cell case had 9812 CD21+ cells/μL with splenomegaly, anemia, and thrombocytopenia, and lymphocytes were large‐sized with prominent nucleoli. The clonal heterogeneous case had abdominal lymphadenopathy and 63 754 CD21+ cells/μL, which were aberrant with decreased CD18 expression by flow cytometry. All B‐cell cases with polyclonal IG rearrangements had <20 000 CD21+ cells/μL, except for 1 case which had 39 700 CD21+ cells/μL with marked neutrophilia (37 000/μL), mild monocytosis, and moderate eosinophilia.

**Figure 3:**
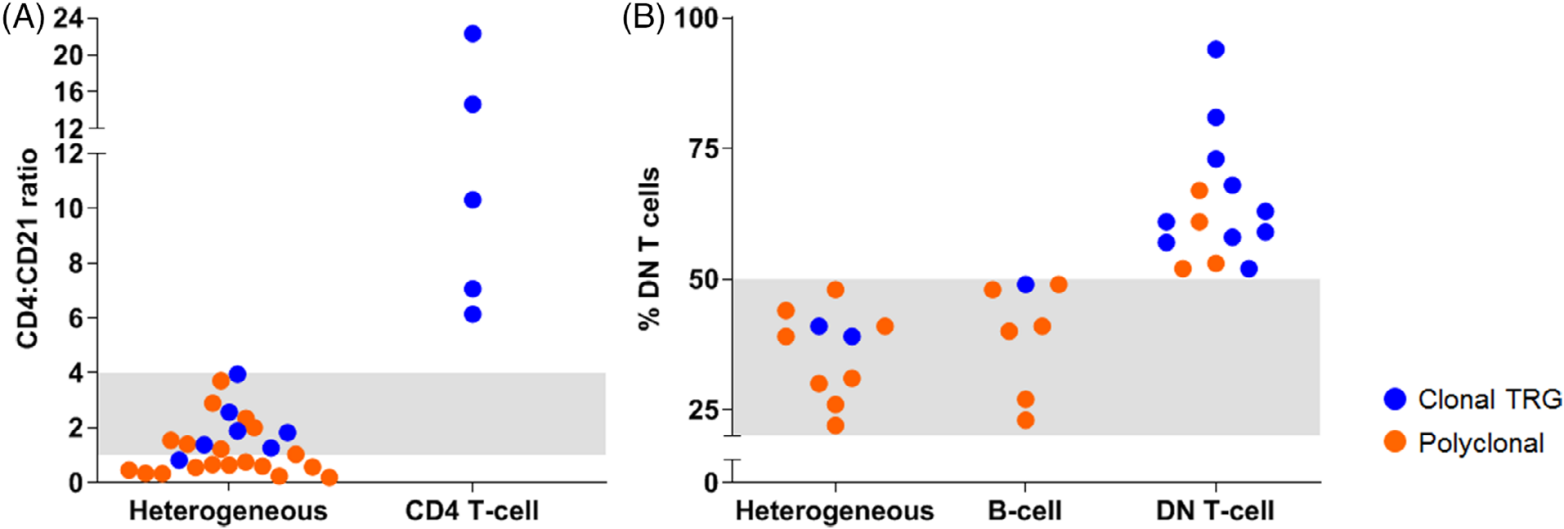
CD4 T‐cell:CD21 B‐cell ratios (A) and DN T‐cell percentages (B) correlated with polymerase chain reaction for antigen receptor rearrangements (PARR) results for cases with flow cytometry and PARR results. These results were used to define flow cytometry phenotypic categories. Cases are colored blue if the sample had a clonal T‐cell receptor gamma (TRG) rearrangement by PARR and orange if TRG rearrangements were polyclonal. A, The CD4 T‐cell:CD21 B‐cell ratio is plotted for cases in the heterogeneous and CD4+ T‐cell groups with dual expansions of CD4+ T cells and CD21+ B cells. Using a ratio of >4 to place cases in the CD4+ T‐cell group, all CD4+ T‐cell cases have a clonal TRG gene rearrangement. Cases within the gray zone, with a ratio between 1 and 4, are comprised of mixed polyclonal (57%) and clonal (43%) TRG results. Eleven of 12 (92%) heterogeneous cases with a CD4:CD21 ratio < 1 were polyclonal. B, The percentage of CD5+ T cells expressing neither CD4 nor CD8 antigens (termed DN T cells) is plotted for cases in heterogeneous, B‐cell, and DN T‐cell groups with >20% DN T cells. Cases with ≥50% DN T cells are classified as DN T cell and 10 of these 14 (71%) cases have a clonal TRG. Cases in the gray zone with 20%‐50% DN T cells had predominantly polyclonal PARR results. Cases with 20% DN T cells were used as the lower threshold of the gray zone because healthy control cats without lymphocytosis had 20%‐25% DN T‐cells. DN, double negative

Two types of cases posed a challenge for classification: (i) cases with heterogeneous expansions, where the magnitude of the CD4+ T‐cell expansion was substantially larger than the B‐cell expansion and (ii) cases with DN T‐cell expansions of small magnitude. In these 2 types of cases, clonality results were used to help refine classification criteria to better segregate non‐neoplastic from neoplastic phenotypes. First, a substantial number of cases (n = 31) had dual expansions of CD4+ T cells and CD21+ B cells. A clonal TRG rearrangement was detected in all cases where the CD4:CD21 lymphocyte ratio was >4 (Figure 3A). Therefore, cases with this ratio subsequently were classified as CD4+ T cell. Clonal TRG rearrangements also were detected in some cases where the CD4:CD21 lymphocyte ratio was <4, but the majority were polyclonal. Therefore, cases with this ratio were put in the heterogeneous group, because it was deemed less harmful to conservatively classify a clinical case as heterogeneous (with a recommendation for PARR confirmation) than to commit it to a neoplastic category of CD4+ T‐cell. Among the heterogeneous cases with CD4:CD21 ratio < 4, the clonal cases were significantly older than the non‐clonal cases (*P* < .001). Six of 7 clonal cases were >7 years old, whereas 17 of 19 non‐clonal cases were <7 years old, suggesting age may be useful in predicting clonality for these cases. All the normal cats used to generate reference intervals had a CD4:CD21 ratio < 1.8. All the cases diagnosed with CD4+ T‐cell lymphocytosis in our study had a CD4:CD21 ratio >4. Second, cases in which ≥50% of the CD5+ T cells expressed neither CD4 nor CD8 antigens were classified as DN T cell. Ten of 14 DN T‐cell cases (71%) had a clonal TRG (Figure 3B) consistent with a neoplastic process. The majority (82%) of cases with 20%‐50% DN T cells, similar to what we documented in control cats, had polyclonal TRG rearrangements, indicating that cats can have up to 50% DN T cells with non‐neoplastic processes.

### 3.3 Clinical presentation

Ninety‐four cats in the outcome cohort were classified into the immunophenotypic categories determined in the definition cohort. Thirty‐nine percent of cases were classified as CD4+ T cells, 18% as heterogeneous, 16% as B cells, 13% as CD5 low T cells, 7.4% as DN T cells, and 5.3% as CD8+ T cells. One case had a homogeneous expansion of CD4+CD8+ T cells, but this phenotype was not seen in any of the other cases in our study, and so this phenotype was not defined as a category and this case was excluded from further analysis. Clinical data for the phenotypic groups are summarized in Table 1.

**Table 1:** Signalment, hematologic findings, and physical examination findings for 93 cats with lymphocytosis in the outcome cohort

	Heterogeneous (n = 17)			B cell (n = 15)			CD8 T cell (n = 5)			CD4 T cell (n = 37)			DN T cell (n = 7)			CD5 low T cell (n = 12)		
	Cats with available data (n)	n	%	Cats with available data (n)	n	%	Cats with available data (n)	n	%	Cats with available data (n)	n	%	Cats with available data (n)	n	%	Cats with available data (n)	n	%
**Signalment**																		
Male	17	6	35.3	15	7	46.7	5	3	60.0	37	17	45.9	7	2	28.6	12	8	66.7
Female	17	11	64.7	15	8	53.3	5	2	40.0	37	20	54.1	7	5	71.4	12	4	33.3
Age, Median (IQR), y	17	5.0 (2.9‐8.9)		15	8.8 (3.5‐13.1)		5	13.8 (8.3‐17.8)		37	12.4 (10.5‐14.8)		7	13.3 (11.5‐14.4)		12	13.8 (9.7‐16.1)	
**Hematologic findings**																		
Lymphocyte total, Median (IQR), ×10^3^/μL	17	16.0 (12.2‐19.5)		15	11.1 (8.5‐14.9)		5	8.9 (6.7‐14.6)		37	28.8 (10.9‐43.5)		7	39.4 (24.5‐132.0)		12	46.6 (18.2‐64.5)	
Anemia (Hematocrit <28)	17	4	23.5	15	6	40.0	5	1	20.0	36	10	27.8	7	3	42.9	12	4	33.3
Thrombocytopenia (<200 000/μL without clumps)	16	2	12.5	15	4	26.7	5	0	0.0	36	9	25.0	7	3	42.9	12	6	50.0
Neutropenia (<2000/μL)	17	1^a^	5.9	15	0	0.0	5	0	0.0	37	2	5.4	7	0	0.0	12	2	16.7
**Physical examination and imaging findings**																		
Peripheral lymphadenopathy	15	1	6.7	13	1	7.7	4	0	0.0	32	2	6.3	7	0	0.0	11	2	18.2
Hepatic abnormalities	14	2	14.3	13	4	30.8	4	0	0.0	27	4	14.8	6	1	16.7	9	2	22.2
Splenic abnormalities	14	2	14.3	13	5	38.5	4	0	0.0	30	12	40.0	5	4	80.0	10	3	30.0
Abdominal lymphadenopathy	11	6	54.5	12	3	25.0	3	2	66.7	28	18	64.3	7	5	71.4	10	4	40.0
Intestinal thickening/mass	12	6	50.0	12	0	0.0	4	2	50.0	26	12	46.2	7	4	57.1	10	4	40.0

*Note*: For all variables except age and lymphocyte count, the number of cases with the particular finding is reported (n), as well as the percent of cases affected among those where data is available within that phenotypic category (%). For age, the median age at diagnosis (in years) and the interquartile range (IQR) are reported for each phenotypic category. For lymphocyte count, the median total lymphocyte count at the time of flow diagnosis (×10^3^/μL) and the IQR are reported. The reference intervals used to determine anemia, thrombocytopenia, and neutropenia are provided. Hepatic and splenic abnormalities included diffuse enlargement, mass effects, or changes in ultrasonographic echogenicity by physical examination, imaging, or both. Intestinal thickening or masses were determined by physical examination, imaging, or both.

a This patient was pancytopenic and positive to feline leukemia virus.

**Figure 4:**
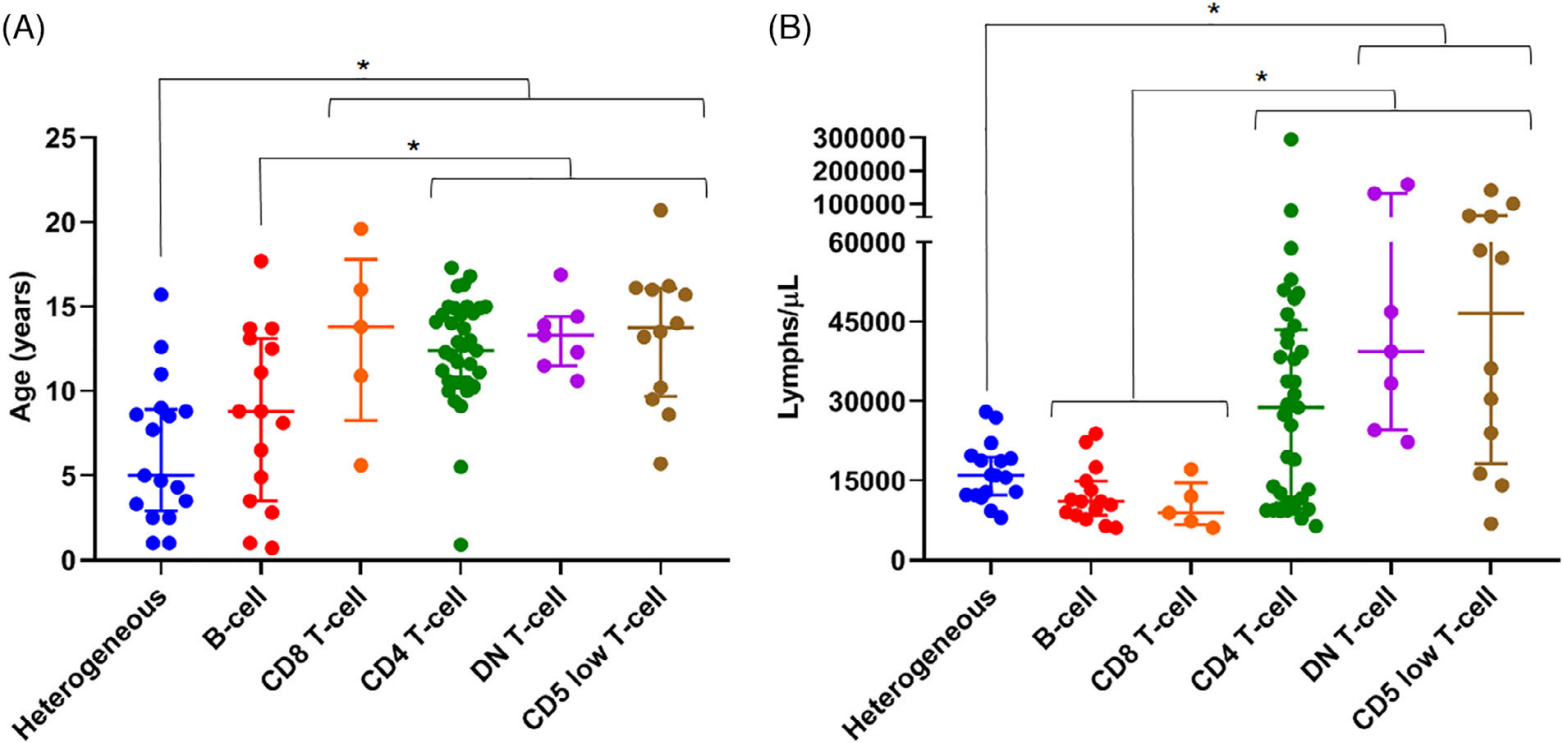
Age (years) (A) and presenting lymphocyte total (lymphs/μL) (B) at time of flow diagnosis for 93 cats by phenotypic group. The median and IQR values for each group are depicted. A, Cats in the heterogeneous group were significantly younger than cats in all 4 of the T‐cell groups (*P* < .02 for each comparison). B‐cell cases were significantly younger than CD4+ T‐cell cases (*P* = .01), DN T‐cell cases (*P* = .03), and CD5 low T‐cell cases (*P* = .01). B, The lymphocyte count was significantly lower in the B‐cell cases and CD8+ T‐cell cases compared to CD4+ T‐cell cases, DN T‐cell cases, and CD5 low T‐cell cases (*P* < .01 for all comparisons). Additionally, heterogeneous cases had lymphocyte counts significantly lower than DN T‐cell cases (*P* = .006) and CD5 low T‐cell cases (*P* = .008). Significant differences are indicated with an asterisk; *P‐value < .05. DN, double negative

Overall median age for cats in the outcome cohort was 11.5 years (interquartile range, [IQR], 8.6‐14.4; range, 1.0‐20.7). The median age for heterogeneous and B‐cell cases was 5.0 and 8.8 years, respectively, whereas the median age was >12.0 years for each of the other groups (Figure 4A). Heterogeneous and B‐cell groups were significantly younger than the neoplastic groups (CD4+ T cell, DN T cell, CD5 low T cell; *P* < .03 for each comparison). Fifty‐three percent of all cases were female, and 47% were male. There was no significant difference in sex between the groups. Breed was reported for 93 cats with 14 breeds represented. Most cats across all groups were domestic shorthair, followed by domestic longhair; remaining breeds were represented in small numbers.

The median lymphocyte count at the time of flow diagnosis for heterogeneous and B‐cell cases was 16 000/μL and 11 100/μL, respectively (Figure 4B). Median lymphocyte counts ranged from 28 800/μL to 46 600/μL among CD4+ T‐cell, DN T‐cell, and CD5 low T‐cell groups. The B‐cell cases had significantly lower counts than did the neoplastic T‐cell groups (*P* < .01 for each comparison), and heterogeneous cases had significantly lower counts than DN and CD5 low T‐cell cases (*P* = .006 and *P* = .008, respectively). Anemia was present in 30% of cases and generally was mild, with a median hematocrit in anemic patients of 24% (IQR, 20‐26; range, 5‐27). Thrombocytopenia was present in 26% of cases. There was no significant difference in anemia or thrombocytopenia among groups. Neutropenia was rare (5.3% of cases, n = 5), and the total neutrophil count was not significantly different among groups. Hypercalcemia was rare, affecting 1 B‐cell case and 1 CD4+ T‐cell case. Hyperglobulinemia also was rare, affecting 2 heterogeneous cases and 1 DN T‐cell case, and serum protein electrophoresis was not performed.

Incidence of peripheral lymphadenopathy, hepatic abnormalities, splenic abnormalities, and abdominal lymphadenopathy was not significantly different among immunophenotypic groups. Intestinal thickening or masses were detected in 39% of cases, and B‐cell cases had significantly less intestinal involvement (none affected) than did all other categories (*P* = .03). Additionally, B‐cell cases (13% affected) had significantly less vomiting or diarrhea reported compared to CD4+ T‐cell cases (62% affected; *P* < .001), DN T‐cell cases (57% affected; *P* = .02), and CD5 low T‐cell cases (33% affected; *P* = .02). Five cats had gastrointestinal histology performed: 3 CD4+ T‐cell cats were diagnosed with intestinal lymphoma, 1 heterogeneous case had lymphoplasmacytic and eosinophilic enteritis, and 1 CD8+ T‐cell case had lymphocytic gastritis with associated helicobacter organisms.

Concurrent diseases were evaluated for B‐cell and heterogeneous cases. Two B‐cell cases and 1 heterogeneous case were positive for FeLV. One B‐cell case was infected with *C felis* and 1 had a *M haemofelis* infection recognized after flow cytometry diagnosis. One heterogeneous case had systemic cryptococcosis. Three cats in this B‐cell/heterogeneous group were diagnosed with IMHA. Other diseases affecting this group included hyperthyroidism (n = 2), pancreatitis (n = 2), urinary disease (n = 3), asthma or allergic disease (n = 2), inflammatory bowel disease (n = 2), and non‐lymphoid neoplasia (n = 1). A small subset of cases had mild neutrophilia, suggesting that a catecholamine stress response may be a differential diagnosis in some cats. Only 2 of the B‐cell cases in this cohort were diagnosed with lymphoma and both were renal‐based. Renal disease, hyperthyroidism, inflammatory bowel disease, and pancreatitis also were reported sporadically in the other immunophenotypes.

### 3.4 Outcome and survival analysis

**Figure 5:**
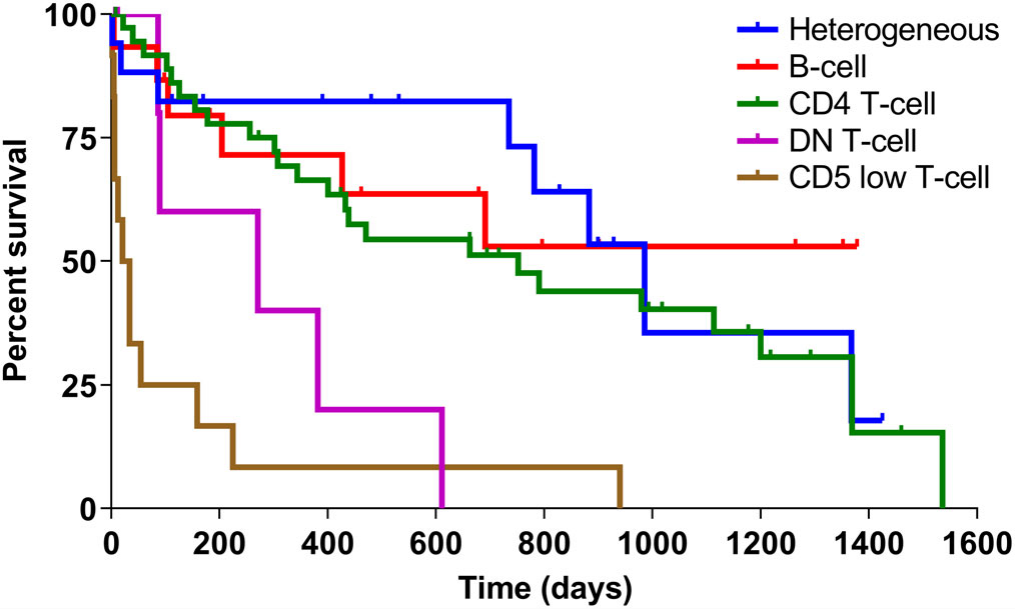
Kaplan‐Meier curves showing overall survival in cats with lymphocytosis of different phenotypes. Adjusting for multiple comparisons, CD5 low T‐cell cases (MST = 27.5 days) had significantly shorter survival than heterogeneous, B‐cell and CD4+ T‐cell cases. DN T‐cell cases (MST = 271 days) had significantly shorter survival than heterogeneous and CD4+ T‐cell cases. CD4+ T‐cell cases (MST = 752 days) and heterogeneous cases (MST = 986 days) had prolonged survival and the MST for B‐cell cases was not reached. DN, double negative

Overall survival from the time of flow diagnosis was examined for all cases in the outcome cohort. Low CD5 expression predicted poor outcome, with CD5 low T‐cell cases having a median survival time (MST) of 27.5 days (range, 2‐940 days; n = 12 cases), which was significantly shorter than heterogeneous, B‐cell, CD8+ T‐cell, and CD4+ T‐cell cases (*P* < .01 for all comparisons; Figure 5). Within the CD5 low T‐cell group, surface expression of CD4 or CD8 did not predict outcome. The DN T‐cell cases (MST, 271 days; range, 7‐611 days; n = 7 cases) had significantly shorter survival compared to heterogeneous (*P* = .01), CD8+ T‐cell (*P* = .04), and CD4+ T‐cell (*P* = .03) cases. The B‐cell cases (MST not reached; n = 15 cases) and heterogeneous cases (MST, 986 days; range, 2‐1425 days; n = 17 cases) had prolonged survival. The CD4+ T‐cell cases had prolonged survival (MST, 752 days; range, 8‐1536 days; n = 37 cases), which was not significantly different from heterogeneous or B‐cell cases. The CD8+ T‐cell cases had a MST of 1217 days (range, 406‐1217 days), but this group only contained 5 cats and 3 of 5 were lost to follow‐up whereas 1 cat died of complications associated with diabetes mellitus. We performed a sensitivity analysis to determine if using the first date of lymphocytosis rather than the date of flow cytometry diagnosis altered our results; our conclusions did not change, except that an MST was now achieved for the B‐cell group (1196 days), and DN T‐cell cases had significantly shorter survival than did the B‐cell cases (*P* = .05).

Factors were assessed for association with survival in the CD4+ T‐cell group because of the size of this cohort and are summarized in Table 2. Sex, abdominal lymph node or intestinal abnormalities or both, and treatment were significantly associated with survival. Female cats (MST, 344 days) had worse outcome compared to males (MST, 1369 days; *P* = .02). There was no difference in survival between patients receiving corticosteroid alone (n = 6) versus corticosteroid and chlorambucil (n = 17), and so these patients were combined in the PO treatment group. Patients receiving combination treatment (MST, 256 days) had shorter survival than did those that received PO treatment alone (MST, 791 days) or no treatment (MST not reached; *P* = .02). The CD4+ T‐cell patients with abdominal lymphadenopathy, intestinal abnormalities, or both had inferior outcome (MST, 471 days) compared to patients without abnormalities detected (MST, 1369 days; *P* = .004).

**Table 2:** Log‐rank test results evaluating potential prognostic factors among CD4+ T‐cell cases

Factor (No. of cases)	MST (days)	95% CI	*P*
Age			
<12 years (n = 16)	1114	471‐NA	.23
≥12 years (n = 21)	439	178‐NA	
Sex			
Female (n = 20)	344	256‐NA	.02^a^
Male (n = 17)	1369	471‐NA	
Lymphocyte count			
<28 800/μL (n = 18)	980	302‐NA	.64
≥28 800/μL (n = 19)	567	401‐NA	
Anemia			
HCT <28 (n = 10)	612	126‐NA	.15
HCT ≥28 (n = 26)	752	401‐NA	
CD4+ T‐cell size^b^			
Small <413 (n = 18)	663	308‐NA	.55
Large ≥413 (n = 18)	752	344‐NA	
Hepatosplenic abnormalities			
Absent (n = 16)	1114	663‐NA	.09
Present (n = 14)	344	126‐NA	
Abdominal lymphadenopathy and/or intestinal abnormalities			
Absent (n = 9)	1369	1200‐NA	.004^a^
Present (n = 20)	471	401‐NA	
Treatment^c^			
None (n = 3)	NA	22‐NA	.02^a^
PO (n = 23)	791	663‐NA	
Injectable/multi‐agent (n = 11)	256	126‐NA	

Abbreviations: CI, confidence interval; HCT, hematocrit; MST, median survival time; NA, not achieved.

a *P*‐value considered statistically different (*P* < .05). Survival times between the groups were tested by log‐rank test.

b Relative CD4+ T‐cell size was determined by geometric mean linear forward scatter. The median size across cases (413) was chosen as a cutoff value to discriminate between small and large cells.

c None: no steroid or chemotherapy treatment given; PO: includes treatment with corticosteroid and/or chlorambucil; Injectable/multi‐agent: includes treatment with single‐agent injectable or multi‐agent chemotherapy protocols.

### 3.5 Concordance with a larger population

**Figure 6:**
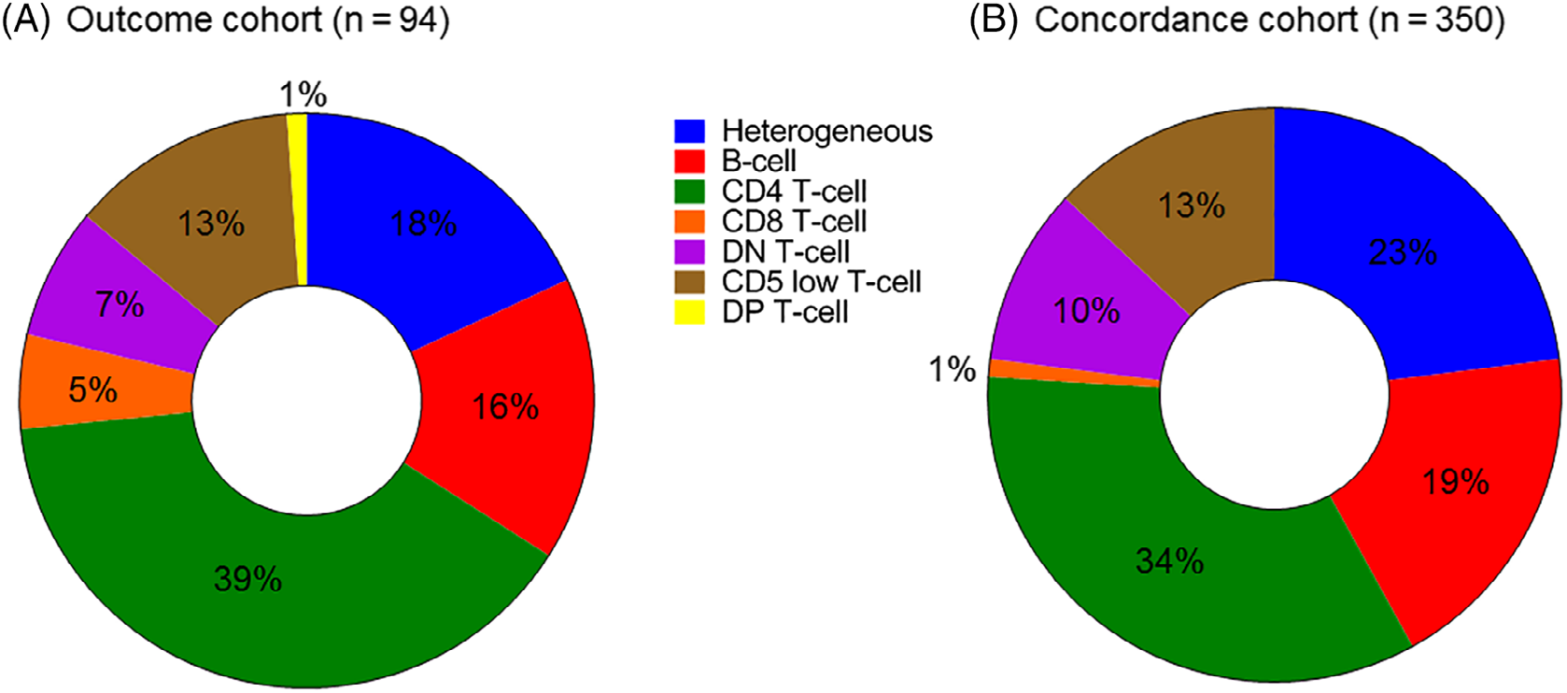
Flow phenotype distribution for the outcome cohort (A) and concordance cohort (B) cats. The distribution of flow phenotypes is presented for all cats in the outcome cohort (n = 94) and the large concordance cohort (n = 350). There were no significant differences between groups, except for the rare CD8+ T‐cell phenotype. The CD4+ T‐cell phenotype was most common in both cohorts, followed by heterogeneous and B‐cell phenotypes. One CD4+CD8+ (DP) T‐cell case was seen in the outcome cohort only. DN, double negative

A concordance cohort of 350 patients was examined to determine whether the composition of the outcome cohort of 94 patients was representative of a larger population. Phenotype distribution, age, and hematologic variables were compared between cohorts. In the large concordance cohort, CD4+ T cell was the most common phenotype (34%, n = 118), followed by heterogeneous (23%, n = 82), B cell (19%, n = 67), CD5 low T cell (13%, n = 47), DN T cell (10%, n = 34), and CD8+ T cell (1%, n = 2; Figure 6). Within the CD5 low T‐cell cases, 55% expressed CD4, 9% expressed CD8, and 36% expressed neither CD4 nor CD8 antigens. When the CD8+ T‐cell phenotype was removed because of the small number of cases, the distribution of phenotypes was not significantly different between the outcome cohort and concordance cohort. The age and sex of patients within each group was not significantly different between the 2 cohorts. The presenting lymphocyte count was higher in the outcome cohort compared to the concordance cohort for DN T‐cell cases only (*P* = .007). Anemia was more common among CD4+ T‐cell cases in the outcome cohort (28% cases affected) compared to the concordance cohort (9% cases affected; *P* = .01), but anemia incidence was not significantly different for other phenotype groups. These findings suggest that the outcome cohort of 94 cats is representative of a larger population.

## 4 DISCUSSION

Our study suggests that non‐neoplastic lymphocytosis is common in cats. Among neoplastic lymphocytosis cases, 3 major T‐cell phenotypes were identified by flow cytometry, and they had different survival times.

Our findings support the hypothesis that cats frequently develop non‐neoplastic lymphocytosis. Both the heterogeneous and B‐cell phenotypes appeared most consistent with non‐neoplastic processes. The major evidence for a non‐neoplastic process in these cases was (i) predominantly polyclonal TRG and IG genes, (ii) younger age at diagnosis, (iii) lower presenting lymphocyte counts, and (iv) prolonged survival. Together, these 2 phenotypes accounted for 42% of the cases in the larger cohort of 350 cats. Expansions of multiple lymphocyte subsets are not typical of true lymphoid neoplasms, and thus it is not surprising that the majority of heterogeneous cases (71%) had polyclonal PARR results. However, it was surprising to see the extent to which cats can have a sole expansion of polyclonal B cells. A relatively pure expansion of 1 subset typically is suggestive of a neoplastic process, but most (89%) of the B‐cell cases were polyclonal and only 2 of these cases had clonal IG rearrangements. We recently have demonstrated that the PARR assay used in our laboratory is 87% sensitive for detecting clonal B‐cell populations. The paucity of clonal IG rearrangements suggests that neoplastic B‐cell lymphocytosis is rare in cats, which corroborates the findings of a previous study.^20^

Many cats with B‐cell or heterogeneous lymphocyte expansions in the outcome cohort had a variety of infectious and inflammatory diseases. The magnitude of the lymphocytosis was striking, in some cases reaching 28 000 lymphocytes/μL. Evidence from experimental Cytauxzoon and Aleurostrongylus infections corroborate these findings where lymphocyte counts up to 31 630 and 36 600 lymphocytes/μL, respectively, were reported.^1^, ^3^ Infections diagnosed in our study cases at the time of flow cytometry diagnosis included *C felis*, FeLV, and cryptococcosis. Three cases (2 heterogeneous, 1 B cell) had apparent immune‐mediated destruction of erythrocytes; these patients were mycoplasma‐negative and the anemia responded to immunosuppression. Interestingly, these cats often maintained the lymphocytosis even while receiving corticosteroids. A review of the literature indicates that, across multiple studies, lymphocytosis has been reported in 15%‐52% of cases of primary IMHA in cats.^10^, ^11^, ^12^, ^13^, ^14^ Lymphocyte counts reaching 20 280 cells/μL have been reported and the 1 case with immunophenotyping had a B‐cell lymphocytosis.^12^ Three cases of IMHA in cats having a mean peripheral lymphocyte count of 14 879 lymphocytes/μL, and increased B cells in the bone marrow were reported. 13 Other conditions identified in cats with B‐cell or heterogeneous expansions in our study included urinary disease, pancreatitis, hyperthyroidism, asthma or other allergic disease, and inflammatory bowel disease.

The propensity for cats to develop non‐neoplastic lymphocytosis created a diagnostic dilemma in some cats with true clonal expansions of T cells. In the definition cohort of 146 cats, 31 cats had expansions of both B cells and CD4+ T cells, and 12 of these 31 cats had clonal TRG rearrangements. We attempted to account for the potential for a non‐neoplastic B‐cell lymphocytosis to accompany a primary clonal T‐cell process by using the ratio of CD4+ T cells:B cells in peripheral blood. Based on our PARR findings, a cutoff of 4:1 helps with this discrimination yet is still imperfect. All cats with a CD4+ T‐cell:B‐cell ratio > 4 (5/5) had clonal TRG rearrangements, whereas 27% of cats with a ratio < 4 (7/26) that were classified as heterogeneous expansions had clonal TRG rearrangements. We felt a conservative cutoff threshold would minimize false‐positive interpretations of neoplasia in a clinical setting. These data suggest that even with an underlying T‐cell neoplasm, cats can mount a secondary non‐neoplastic B‐cell lymphocytosis, and PARR is strongly recommended in these cases to ultimately confirm clonality.

The most common phenotype in our study, CD4+ T‐cell lymphocytosis, had an indolent clinical course consistent with previous reports.^20^, ^30^ We found that treatment type, intra‐abdominal organ involvement, and sex influenced survival in CD4+ T‐cell cases. The effect of treatment on survival is difficult to assess in a retrospective analysis, because treatment decisions can be influenced by the severity of the presenting illness or the owners' willingness to pursue treatment. The apparent shorter survival in the injectable/multi‐agent chemotherapy group may simply reflect that these cats presented with more advanced disease, or adverse effects associated with a more intensive treatment protocol may have decreased quality of life and potentially led to death or euthanasia. Alternatively, it is possible that the mechanism of action for chlorambucil is more efficacious in treating CD4+ T‐cell lymphocytosis than is a multi‐drug protocol, as has been suggested for slowly dividing well‐differentiated tumors such as low‐grade alimentary lymphoma in cats.^31^, ^32^ The overall survival of 791 days in cats treated with corticosteroid, chlorambucil, or both suggests this regimen is an appropriate treatment for many cats with CD4+ T‐cell lymphocytosis. Although we did not detect a survival difference between cats treated with corticosteroid alone versus corticosteroid and chlorambucil, very few cats were treated with corticosteroid alone. Determination of optimal treatment protocols would require larger, prospective studies. Detectable abdominal lymphadenopathy or intestinal abnormalities or both were associated with shorter survival time in cats with CD4+ T‐cell lymphocytosis, which may represent a more advanced stage of disease or a different disease process. Female CD4+ T‐cell had shortened survival compared to males. A retrospective study of gastric lymphoma in cats found that females had shorter survival compared to males,^33^ suggesting sex may be prognostic in certain lymphoproliferative disorders of cats. Age, anemia, presenting lymphocyte count, and cell size appear to influence outcome in some forms of lymphoma or leukemia in dogs,^34^, ^35^, ^36^ but were not associated with survival in CD4+ T‐cell lymphocytosis of cats. Limitations of this outcome study include its retrospective design, duration of time from case management to data collection, and incomplete staging for some cases.

The proportion of cats in our study with intestinal involvement was notable. Forty to 57% of heterogeneous, CD4+ T‐cell, DN T‐cell, and CD5 low T‐cell cases had intestinal abnormalities detected by palpation or imaging. Among cases with intestinal histopathology performed, 1 heterogeneous case was diagnosed as inflammatory bowel disease and 3 CD4+ T‐cell cases were diagnosed as intestinal lymphoma. These findings suggest that cats with intestinal abnormalities and concurrent lymphocytosis may have non‐neoplastic or neoplastic intestinal diseases. One study describing both intestinal lymphoma and chronic enteritis also reported increased lymphocyte counts in cats from both categories.^22^ In our study, 12 cats with CD4+ T‐cell lymphocytosis in the outcome cohort had both clinical signs and ultrasonographic evidence of gastrointestinal disease at the time of diagnosis. Interestingly, another study documented mesenteric lymphadenopathy in 9 of 13 and intestinal thickening in 3 of 13 cats included in a group of cats with clinically defined CLL and a predominant CD4+ T‐cell phenotype.^20^ Additionally, 2 cats were described as having concurrent low‐grade intestinal lymphoma.^20^ Although it currently is unknown if cases of low‐grade alimentary lymphoma in cats typically are comprised of CD4+ T cells, our limited histology data suggest that some of our CD4+ T‐cell lymphocytosis cases had an intestinal tissue component.

Our results indicate that cats presenting with aberrant phenotypes, in this case increased DN T cells or low CD5 expression, had significantly shorter survival times, but the number of cases was small. The MST of 27.5 days in the CD5 low T‐cell group was particularly notable. Interestingly, in a study of LGL lymphoma with peripheral lymphocytosis, 15 of 21 cases were described as CD5− and had variable expression of CD8 and CD4 antigens.^26^ Compiling the 63 CD5 low T‐cell cases from all 3 of our cohorts, 51% expressed CD4, 38% were DN, and 11% expressed CD8. With only 12 CD5 low T‐cell cases in the outcome cohort, we did not detect differences in clinical presentation or outcome based on CD4 or CD8 expression, and all CD5 low cases were grouped together. However, a future study with larger numbers of cases may determine that the CD5 low T‐cell group encompasses a mixture of neoplasms.

A notable feature of our case series is the relative extent to which the DN T‐cell population can expand in cats with both neoplastic and non‐neoplastic conditions. Using the antibodies in our flow cytometry panel, we routinely can detect small numbers of circulating CD5+CD4/8− cells in normal cats (up to 1000 cells/μL). Because the simple presence of this phenotype could not be used to classify a sample as neoplastic, we utilized the definition cohort to delineate a cutoff of >50% DN T cells. Similar to the ratio of CD4+ T cells:B cells, this cutoff was chosen to conservatively classify cases as neoplastic. Polymerase chain reaction for antigen receptor rearrangements remains a necessary clinical follow‐up test in any cat with substantially expanded DN T cells. In a small number of PARR‐negative B‐cell and heterogeneous cases, DN cells accounted for as many as 49% of all T cells, although the absolute numbers of DN T cells remained relatively modest, demonstrating the extent to which the proportion of non‐neoplastic DN T cells may be expanded. Neoplastic changes resulting in the loss of surface molecule expression are relatively common in lymphomas and leukemias,^37^ but the origin of this DN population in non‐neoplastic states is less clear. We frequently identify a small population of CD8 low T cells in cat blood as has been reported by others,^38^, ^39^, ^40^ but after accounting for these, a true DN T‐cell population still is detected. One possibility for a lymphocyte expressing CD5 or CD3 but lacking CD4/CD8 expression would be γδT cells. In cows, sheep, and pigs, γδT cells can comprise the majority of peripheral blood mononuclear cells (PBMC) in young animals and typically decrease to 8%‐18% and 15%‐35% of adult bovine and porcine PBMC, respectively.^41^, ^42^, ^43^, ^44^ Such γδT cells have not been specifically identified in cats and detailed descriptions of feline DN T‐cells are lacking, although many studies have not included pan‐T‐cell markers when analyzing CD4+ and CD8+ T‐cell subsets. More detailed analysis of this feline lymphocyte subset is warranted to determine its origin.

In summary, our study highlights some of the challenges in characterizing lymphocytosis in cats, especially given the propensity for dual expansions of lymphocyte subsets. Although flow cytometry alone was diagnostic in many cases, a subset still required PARR to distinguish a non‐neoplastic from neoplastic process. We found that cats frequently have heterogeneous lymphocyte and sole B‐cell expansions in the blood, which are predominantly non‐neoplastic in nature. Among the neoplastic phenotypes identified, CD4+ T‐cell, DN T‐cell, and CD5 low T‐cell cases had prolonged, intermediate, and short MSTs, respectively. Flow cytometry is a useful tool to differentiate these T‐cell phenotypes with highly variable clinical courses.

## CONFLICT OF INTEREST DECLARATION

Authors declare no conflict of interest.

## OFF‐LABEL ANTIMICROBIAL DECLARATION

Authors declare no off‐label use of antimicrobials.

## INSTITUTIONAL ANIMAL CARE AND USE COMMITTEE (IACUC) OR OTHER APPROVAL DECLARATION

This retrospective study used materials submitted for diagnostic purposes and information in medical records.

## HUMAN ETHICS APPROVAL DECLARATION

Authors declare human ethics approval was not needed for this study.

## Supporting information


**Appendix**S1: Supporting Information1

## References

[1] Reichard M , Meinkoth JH , Edwards AC , et al. Transmission of Cytauxzoon felis to a domestic cat by Amblyomma americanum. Vet Parasitol. 2009;161(1–2):110‐115. 19168288 10.1016/j.vetpar.2008.12.016

[2] Lappin MR , George JW , Pedersen NC , Barlough JE , Murphy CJ , Morse LS . Primary and secondary Toxoplasma gondii infection in normal and feline immunodeficiency virus‐infected cats. J Parasitol. 1996;82(5):733‐742. 8885881

[3] Schnyder M , Di Cesare A , Basso W , et al. Clinical, laboratory and pathological findings in cats experimentally infected with Aelurostrongylus abstrusus. Parasitol Res. 2014;113(4):1425‐1433. 24504600 10.1007/s00436-014-3783-2

[4] Ayllon T , Tesouro MA , Amusategui I , Villaescusa A , Rodriguez‐Franco F , Sainz Á . Serologic and molecular evaluation of Leishmania infantum in cats from Central Spain. Ann N Y Acad Sci. 2008;1149:361‐364. 19120250 10.1196/annals.1428.019

[5] Harrus S , Klement E , Aroch I , et al. Retrospective study of 46 cases of feline haemobartonellosis in Israel and their relationships with FeLV and FIV infections. Vet Rec. 2002;151(3):82‐85. 12164225 10.1136/vr.151.3.82

[6] Gleich S , Hartmann K . Hematology and serum biochemistry of feline immunodeficiency virus‐infected and feline leukemia virus‐infected cats. J Vet Intern Med. 2009;23(3):552‐558. 19645840 10.1111/j.1939-1676.2009.0303.x

[7] Sparkes AH , Hopper CD , Millard WG , Gruffydd‐Jones TJ , Harbour DA . Feline immunodeficiency virus infection clinicopathologic findings in 90 naturally occurring cases. J Vet Intern Med. 1993;7(2):85‐90. 8388953 10.1111/j.1939-1676.1993.tb03174.x

[8] Raimundo JM , Guimarães A , Botelho CFM , et al. Hematological changes associated with hemoplasma infection in cats in Rio de Janeiro, Brazil. Rev Bras Parasitol Vet. 2016;25(4):441‐449. 27982300 10.1590/S1984-29612016086

[9] Breitschwerdt EB , Levine JF , Radulovic S , Hanby SB , Kordick DL . Perle KMD La, et al. Bartonella henselae and rickettsia seroreactivity in a sick cat population from North Carolina. Int J Appl res. Vet Med. 2005;3:287‐302.

[10] Husbands BD , Smith SA , Weiss DJ. Idiopathic immune‐mediated hemolytic anemia (IMHA) in 25 cats. In: 20th Annual ACVIM Forum, 2002, p. 33.

[11] Kohn B , Weingart C , Eckmann V , Ottenjann M , Leibold W . Primary immune‐mediated hemolytic anemia in 19 cats: diagnosis, therapy, and outcome (1998‐2004). J Vet Intern Med. 2006;20(1):159‐166. 16496936 10.1892/0891-6640(2006)20[159:pihaic]2.0.co;2

[12] Swann JW , Szladovits B , Glanemann B . Demographic characteristics, survival and prognostic factors for mortality in cats with primary immune‐mediated hemolytic anemia. J Vet Intern Med. 2016;30(1):147‐156. 26645865 10.1111/jvim.13658 PMC4913623

[13] Weiss DJ . Differentiating benign and malignant causes of lymphocytosis in feline bone marrow. J Vet Intern Med. 2005;19(6):855‐859. 16355680 10.1892/0891-6640(2005)19[855:dbamco]2.0.co;2

[14] Weiss DJ . Bone marrow pathology in dogs and cats with non‐regenerative immune‐mediated haemolytic anaemia and pure red cell aplasia. J Comp Pathol. 2008;138(1):46‐53. 18083185 10.1016/j.jcpa.2007.10.001

[15] Peterson ME , Kintzer PP , Hurvitz AI . Methimazole treatment of 262 cats with hyperthyroidism. J Vet Intern Med. 1988;2(3):150‐157. 3265728 10.1111/j.1939-1676.1988.tb02812.x

[16] Thoday KL , Mooney CT . Historical, clinical and laboratory features of 126 hyperthyroid cats. Vet Rec. 1992;131(12):257‐264. 1413411 10.1136/vr.131.12.257

[17] Frénais R , Rosenberg D , Burgaud S , Horspool LJI . Clinical efficacy and safety of a once‐daily formulation of carbimazole in cats with hyperthyroidism. J Small Anim Pract. 2009;50(10):510‐515. 19796309 10.1111/j.1748-5827.2009.00772.x

[18] Peterson ME , Greco DS , Orth DN . Primary Hypoadrenocorticism in ten cats. J Vet Intern Med. 1989 Apr 1;3(2):55‐58. 2469793 10.1111/j.1939-1676.1989.tb03080.x

[19] Tasker S , MacKay AD , Sparkes AH . A case of feline primary Hypoadrenocorticism. J Feline Med Surg. 1999 Dec 24;1(4):257‐260. 11714243 10.1053/jfms.1999.0044 PMC10822371

[20] Campbell MW , Hess PR , Williams LE . Chronic lymphocytic leukaemia in the cat: 18 cases (2000‐2010). Vet Comp Oncol. 2013;11(4):256‐264. 22372648 10.1111/j.1476-5829.2011.00315.x

[21] Tomiyasu H , Doi A , Chambers JK , et al. Clinical and clinicopathological characteristics of acute lymphoblastic leukaemia in six cats. J Small Anim Pract. 2018 Dec;59(12):742‐746. 30168590 10.1111/jsap.12917

[22] Norsworthy GD , Estep JS , Hollinger C , et al. Prevalence and underlying causes of histologic abnormalities in cats suspected to have chronic small bowel disease: 300 cases (2008–2013). J Am Vet Med Assoc. 2015;247(6):629‐635. 26331421 10.2460/javma.247.6.629

[23] Pope K , Tun AE , McNeill CJ , Brown DC , Krick EL . Outcome and toxicity assessment of feline small cell lymphoma: 56 cases (2000‐2010). Vet Med Sci. 2015;1(2):51‐62. 29067174 10.1002/vms3.9 PMC5645816

[24] Gabor LJ , Canfield PJ , Malik R . Haematological and biochemical findings in cats in Australia with lymphosarcoma. Aust Vet J. 2000;78(7):456‐461. 10923176 10.1111/j.1751-0813.2000.tb11856.x

[25] Wellman ML , Hammer AS , DiBartola SP , Carothers MA , Kociba GJ , Rojko JL . Lymphoma involving large granular lymphocytes in cats: 11 cases (1982‐1991). J Am Vet Med Assoc. 1992;201(8):1265‐1269. 1429172

[26] Roccabianca P , Vernau W , Caniatti M , Moore PF . Feline large granular lymphocyte (LGL) lymphoma with secondary leukemia: primary intestinal origin with predominance of a CD3/CD8aa phenotype. Vet Pathol. 2006;43(1):15‐28. 16407483 10.1354/vp.43-1-15

[27] Krick EL , Little L , Patel R , et al. Description of clinical and pathological findings, treatment and outcome of feline large granular lymphocyte lymphoma (1996‐2004). Vet Comp Oncol. 2008;6(2):102‐110. 19178669 10.1111/j.1476-5829.2007.00146.x

[28] Finotello R , Vasconi ME , Sabattini S , et al. Feline large granular lymphocyte lymphoma: an Italian Society of Veterinary Oncology (SIONCOV) retrospective study. Vet Comp Oncol. 2018;16(1):159‐166. 28556532 10.1111/vco.12325

[29] Rout ED , Burnett RC , Yoshimoto JA , Avery PR , Avery AC . Assessment of immunoglobulin heavy chain, immunoglobulin light chain, and T‐cell receptor clonality testing in the diagnosis of feline lymphoid neoplasia. Vet Clin Pathol. 2019;48(S1):45‐58. 31478220 10.1111/vcp.12767

[30] Workman HC , Vernau W . Chronic lymphocytic leukemia in dogs and cats: the veterinary perspective. Vet Clin North Am Small Anim Pract. 2003;33(6):1379‐1399. 14664204 10.1016/s0195-5616(03)00120-7

[31] Lingard AE , Briscoe K , Beatty JA , et al. Low‐grade alimentary lymphoma: clinicopathological findings and response to treatment in 17 cases. J Feline Med Surg. 2009;11(8):692‐700. 19576832 10.1016/j.jfms.2009.05.021 PMC11132580

[32] Fondacaro JV , Richter KP , Carpenter JL , Hart JR , Hill SL , Fettman MJ . Feline gastrointestinal lymphoma: 67 cases (1988‐1996). Eur J Comp Gastroenterol. 1999;4(2):5‐11.

[33] Gustafson TL , Villamil A , Taylor BE , Flory A . A retrospective study of feline gastric lymphoma in 16 chemotherapy‐treated cats. J Am Anim Hosp Assoc. 2014;50(1):46‐52. 24216491 10.5326/JAAHA-MS-5989

[34] Comazzi S , Gelain ME , Martini V , et al. Immunophenotype predicts survival time in dogs with chronic lymphocytic leukemia. J Vet Intern Med. 2011;25(1):100‐106. 21092008 10.1111/j.1939-1676.2010.0640.x

[35] Williams MJ , Avery AC , Lana SE , Hillers KR , Bachand AM , Avery PR . Canine lymphoproliferative disease characterized by lymphocytosis: Immunophenotypic markers of prognosis. J Vet Intern Med. 2008;22(3):596‐601. 18346150 10.1111/j.1939-1676.2008.0041.x

[36] Rao S , Lana S , Eickhoff J , et al. Class II major histocompatibility complex expression and cell size independently predict survival in canine B‐cell lymphoma. J Vet Intern Med. 2011;25(5):1097‐1105. 21781170 10.1111/j.1939-1676.2011.0767.x

[37] Rydzewski L , Scheffold S , Hecht W , et al. Identification of a novel feline large granular lymphoma cell line (S87) as non‐MHC‐restricted cytotoxic T‐cell line and assessment of its genetic instability. Vet Immunol Immunopathol. 2016;177:24‐34. 27436441 10.1016/j.vetimm.2016.05.012

[38] Shimojima M , Pecoraro MR , Maeda K , Tohya Y , Miyazawa T , Mikami T . Characterization of anti‐feline CD8 monoclonal antibodies. Vet Immunol Immunopathol. 1998;61:17‐23. 9613469 10.1016/s0165-2427(97)00091-3

[39] Willett BJ , Hosie MJ , Callanan JJ , Neil JC , Jarrett O . Infection with feline immunodeficiency virus is followed by the rapid expansion of a CD8+ lymphocyte subset. Immunology. 1993;78(1):1‐6. 8094707 PMC1421779

[40] Miranda LHM , Santiago MA , Schubach TMP , et al. Severe feline sporotrichosis associated with an increased population of CD8 ^low^ cells and a decrease in CD4 ^+^ cells. Med Mycol. 2016;54(1):29‐39. 26483429 10.1093/mmy/myv079

[41] Mackay CR , Hein WR . A large proportion of bovine T cells express the gamma delta T cell receptor and show a distinct tissue distribution and surface phenotype. Int Immunol. 1989;1(5):540‐545. 2535142 10.1093/intimm/1.5.540

[42] Mackay CR , And JFM , Brandon MR . Three distinct subpopulations of sheep T lymphocytes. Eur J Immunol. 1986;16(1):19‐25. 3081353 10.1002/eji.1830160105

[43] Takamatsu HH , Denyer MS , Stirling C , et al. Porcine γδ T cells: possible roles on the innate and adaptive immune responses following virus infection. Vet Immunol Immunopathol. 2006;112(1–2):49‐61. 16714063 10.1016/j.vetimm.2006.03.011

[44] Rogers AN , VanBuren DG , Hedblom EE , et al. Cell function varies with the expressed WC1 coreceptor. J Immunol. 2005;174(6):3386‐3393. 15749871 10.4049/jimmunol.174.6.3386

