# Supplementary material for: Immunophenotypic characterization and clinical outcome in cats with lymphocytosis
Source: J Vet Intern Med. 2019 Nov 6;34(1):105–16. doi: 10.1111/jvim.15650 (PMC6979106; doi:10.1111/jvim.15650)
Supplement: Supplementary file 1 — AppendixS1: Supporting Information1 [file JVIM-34-105-s001.pdf]

Supplemental material – flow cytometry methods and antibody panel

Flow cytometry methods

Blood sample collection, storage and red cell lysis were performed as described in Rout et al., 2019.[1] Resulting cell pellets were stained as previously described, using antibody solution provided in supplemental table 1. For all samples, propidium iodide staining was used to exclude dead cells during analysis.

All of the samples in the outcome cohort were received before May 8, 2012 and were stained with antibody panel 1 in supplemental table 1 and analyzed on a single-laser Coulter XL flow cytometer. All of the samples in the definition cohort and concordance cohort were received after October 8, 2014, and were stained with antibody panel 2 in supplemental table 1 and analyzed on a 3-laser Coulter Gallios flow cytometer. Data analysis was carried out with Kaluza software (Beckman Coulter, Brea, CA).

Supplemental table 1. Antibody panels used for feline flow cytometric immunophenotyping.

Tube	Antibody specificity and fluorochrome
Panel 1 (two color)	
1	None
2	CD14-FITC / Mouse IgG1-PE
3	Mouse IgG1-FITC / CD21-PE
4	CD4-FITC / CD8-PE
5	CD5-FITC / CD21-PE
6	CD18-FITC / CD14-PE

Panel 2 (multicolor)	
1	None
2	CD4-FITC / CD8-PE / CD18-Alexa 647 / CD61-PO*
3	CD5-FITC / CD21-PE / CD18-Alexa 647 / CD61-PO*

Clones are as follows: CD14 = UCHM1 (human), CD21 = CA2.1D6 (canine), CD4 = vpg34 (feline), CD8 = vpg9 (feline), CD5 = FE1.1B11 (feline), panel 1 CD18 = YFC118.3 (human), panel 2 CD18 = CA1.4E9 (canine), CD61 = VI-PL2 (human).

*The CD61 antibody was conjugated using a Pacific Orange antibody labeling kit (Thermo Fisher, Waltham, MA). All other antibodies were purchased directly conjugated.

All antibodies were purchased from Bio-Rad, Hercules, CA, except for the CD61 antibody, which was purchased from BD Biosciences, San Jose, CA.

Each antibody tube (25 µL) was combined with 25 µL sample in a single well of a 96-well plate.

Reference

1. Rout ED, Burnett RC, Yoshimoto JA, Avery PR, Avery AC. Assessment of immunoglobulin heavy chain, immunoglobulin light chain, and T- cell receptor clonality testing in the diagnosis of feline lymphoid neoplasia. Vet Clin Pathol. 2019;48(S1):45–58.
